# The Impact of Gastrointestinal Symptoms and Dermatological Injuries on Nutritional Intake and Hydration Status During Ultramarathon Events

**DOI:** 10.1186/s40798-015-0041-9

**Published:** 2016-01-05

**Authors:** Ricardo J. S. Costa, Rhiannon Snipe, Vera Camões-Costa, Volker Scheer, Andrew Murray

**Affiliations:** 1 Department of Nutrition & Dietetics, Monash University, Notting Hill, Victoria Australia; 2 British Forces Germany Health Services, Paderborn, Germany; 3 Centre of Sports & Exercise, Edinburgh University, Edinburgh, UK

## Abstract

**Background:**

Debilitating gastrointestinal symptoms (GIS) and dermatological injuries (DI) are common during and after endurance events and have been linked to performance decrements, event withdrawal, and issues requiring medical attention. The study aimed to determine whether GIS and DI affect food and fluid intake, and nutritional and hydration status, of ultramarathon runners during multi-stage (MSUM) and 24-h continuous (24 h) ultramarathons.

**Methods:**

Ad libitum food and fluid intakes of ultramarathon runners (MSUM *n* = 54; 24 h *n* = 22) were recorded throughout both events and analysed by dietary analysis software. Body mass and urinary ketones were determined, and blood samples were taken, before and immediately after running. A medical log was used to monitor symptoms and injuries throughout both events.

**Results:**

GIS were reported by 85 and 73 % of ultramarathon runners throughout MSUM and 24 h, respectively. GIS during MSUM were associated with reduced total daily, during, and post-stage energy and macronutrient intakes (*p* < 0.05), whereas GIS during 24 h did not alter nutritional variables. Throughout the MSUM 89 % of ultramarathon runners reported DI. DI during MSUM were associated with reduced carbohydrate (*p* < 0.05) intake during running and protein intake post-stage (*p* < 0.05). DI during 24 h were low; thus, comparative analyses were not possible. Daily, during running, and post-stage energy, macronutrient and water intake variables were observed to be lower with severity of GIS and DI (*p* < 0.05) throughout the MSUM only.

**Conclusions:**

GIS during the MSUM, but not the 24 h, compromised nutritional intake. DI presence and severity also compromised nutrient intake during running and recovery in the MSUM.

## Key Points

- Gastrointestinal symptoms (GIS) were a common feature in both the MSUM and 24 h; however, dermatological injuries (DI) were high during the MSUM, but relatively low during the 24 h.
- Incidence and severity of GIS appeared to be associated with greater disturbances to daily energy balance, reduced daily macronutrient intake, and reduced nutritional intake during running and recovery throughout the MSUM, but not the 24 h.
- Incidence and severity of DI appeared to be associated with reduced nutritional intake during running and recovery throughout the MSUM.

## Background

Ultramarathon running events (>42.195 km) have increased in popularity over the past decade [1] and are predicted for future growth within recreational endurance sports participation. Despite popularity, research into the demands of such an extreme sport is scarce in comparison to shorter endurance running events (e.g. marathon and sub-marathon) [2]. Participation in ultra-endurance events often requires competitors to perform loaded prolonged physical exertion, sleep rough on consecutive days, and manage food and fluid provisions throughout competition to maintain performance and avoid adverse health outcomes [3, 4]. Such diverse challenges demonstrates the uniqueness of extreme endurance sports and highlights areas for potential performance decrements and health issues to exacerbate.

Extreme energy deficits are a common feature of continuous and multi-stage ultramarathon running events and have been associated with poor recovery quality and sustained fatigue [5, 6]. Development of unintentional symptoms (e.g. gastrointestinal) and injury (e.g. dermatological) may limit total food and fluid intake during consecutive days of exertional stress with and without the additional impact of environmental extremes [3, 7]. Moreover, sub-optimal nutrition and hydration during periods of extreme exertion is clinically relevant, having been implicated in exacerbating exercise-induced immunodepression, impaired repair and healing, cytokine dysfunction associated with chronic fatigue, rapid onset microcytic anaemia, exercise-induced rhabdomyolysis, and exertional heat illnesses [3, 8–13].

Gastrointestinal symptoms (GIS) are a common feature of endurance sports and appear to be more pronounced in running compared to other exercise modes [14] and greater in a dehydrated state compared with a euhydrated state [15]. Indeed, 93 % of a participant cohort reported GIS during a long distance triathlon event, with upper-GIS (e.g. belching, bloating, and vomiting) and nausea reported to predominate [16]; whereas 60 % of ultramarathon runners during a continuous 161-km ultramarathon reported upper- (e.g. vomiting) and lower (e.g. abdominal cramps and diarrhoea)-GIS, with 89 % of participants with symptoms also reporting nausea [17]. Gastrointestinal symptoms (e.g. nausea and vomiting) have also been reported to be a key contributing factor in performance decrements and event withdrawal during ultramarathon running competition [18, 19].

The causes of GIS during prolonged exertion are multi-factorial in origin. Splanchnic hypoperfusion, ischaemia, alterations to intestinal motility, and mechanical trauma appear to play a key role [15, 20, 21]. When exertion is performed in hot environmental conditions (>30 °C), enhanced thermoregulatory strain, increased body water losses, and accompanying hypovolaemia promote further splanchnic hypoperfusion, ischaemia, and disruption of intestinal epithelial integrity [22–24]. Such perturbations have been linked to increased epithelial permeability of bacterial endotoxins, originating from the intestinal microbiota, leading to endotoxaemia and subsequent responsive cytokinaemia [25, 26], in which nausea is a common feature. Moreover, nausea and (or) vomiting has also been reported during episodes of heat illness [27], asymptomatic and symptomatic exercise-associated hyponatremia (with or without encephalopathy) [28, 29], and may occur if ultramarathon runners attempt to meet high energy and water demands through constant forced feeding and stimulation of the ileal brake [30].

Dermatological injuries (DI) (e.g. blisters, subungual haematoma, chafing, abrasions, and sunburn) are also a common feature of endurance sports [4, 31–33]. Dermatological injuries account for the greatest number of medical interventions and event withdrawals during multi-stage ultramarathon events [7, 33, 34] and the prime factor to adversely affect performance in continuous ultramarathon competition [18, 35]. The main causes of such injuries are excessive frictional forces and surface exposure, which are exacerbated by heat, poorly-fitted clothing and equipment, carrying load, and individual characteristics (i.e. skin surface roughness) [33, 36]. An anecdotal observational case scenario from the seven-stages 245 km 2008 Marathon des Sables indicated that all members (*n* = 6) of tent 96 developed foot injuries by stage 2, with injury numbers and severity increasing thereafter. All members reported investing a substantial amount of time with injury care and pain management (i.e. 2–3 h during recovery; including self-management, awaiting and receiving medical attention), deterring focus and priority away from scheduled feeding regimes, and potentially creating a barrier towards meeting high nutritional and hydration requirement, which can substantially effect running performance.

To date, the impact of GIS and DI on nutritional intake and hydration status during continuous ultramarathon events longer than 161 km, and multi-stage ultramarathon events, has not been thoroughly investigated. The purpose of this study was to determine whether GIS or DI affects food and fluid intake, and nutrition and hydration status, of ultramarathon runners during a multi-stage ultramarathon and a 24-h continuous ultramarathon.

## Methods

### Ultramarathon Events and Participants

The multi-stage ultramarathon (MSUM) component of the study was conducted during the 2010 and 2011 Al Andalus Ultimate Trail (*www.alandalus-ut.com*), held during the second week of July, in the region of Loja, Spain (Table 1). The 24-h continuous ultramarathon (24 h) component of the study was conducted during the 2011 and 2012 Glenmore24 Trail Race (*http://runyabam.com/glenmore-24/*), held during the first week of September, in the Cairngorms National Park, Scottish Highlands, UK.

**Table 1:** Participant and event characteristics of the multi-stage (MSUM) and 24-h continuous (24 h) ultramarathons

	MSUM	24 h
Participant characteristics		
Total (*n*)	54	22
Male	33	16
Female	21	6
Age (year)	40 ± 8	40 ± 7
Height (m)	171 ± 15	177 ± 8
Body mass (kg)	70 ± 11	78 ± 11
Number of previous ultramarathon events	5 ± 7	16 ± 13
Ultramarathon characteristics		
Distance (km)	total 225	range 122 to 208
Ambient temperature (°C)	range 32 to 40	range 0 to 20
Relative humidity (% range)	range 32 to 40	range 54 to 82
Altitude (m)	range 473 to 1443	range 39 to 645
Course description	Off-road trails and paths, steep and narrow mountain passes, and occasional road	Off-road terrains, including trails, paths, and grasslands

Mean ± SD (otherwise specified)

After ethical approval from the University Ethics Committee that conforms with the Helsinki declaration for human research ethics, 74 out of 134 ultramarathon runners entered in the MSUM event, and 25 out of 48 ultramarathon runners entered in the 24 h event provided informed consent to participate in the study. However, complete gastrointestinal and dermatological profiles and nutritional and hydration variables were obtained in *n* = 54 and *n* = 22 for the MSUM and 24 h events, respectively (Table 1). Only participants who presented complete gastrointestinal and dermatological profiles and nutritional and hydration variables were used in comparative data analysis.

### Study Design and Data Collection

Both ultramarathons were self-sufficient, whereby participants planned and provided their own ad libitum foods and fluids along the duration of the events. Only plain water was provided by the race organisers. Aid stations along the running course were situated approximately 10 and 3 km apart for the MSUM and 24 h, respectively. For the MSUM, estimated total daily energy expenditure was calculated through predictive equations [37] and verified through SenseWear (7.0, BodyMedia Inc., Pittsburgh, PA, USA) in a sub-sample of ultramarathon runners using convenience sampling (22 verifications, coefficient of variation 5.6 %), as previously used to guide sports dietetic support during MSUM competition [38]. For the 24 h, estimated total energy expenditure was determined by SenseWear in all participants. The triaxial accelerometry, which also included measurements of heat flux, skin temperature, and galvanic skin responses, was attached firmly to the upper arm of the participants, over the mid-point of the triceps muscle, during the measurement period. Data were processed using proprietary algorithms available in the software (version 7.0, algorithm version 2.2.4).

Before the start of each event (and each stage of the MSUM), participants were asked to provide a mid-flow urine sample into 30 ml universal tubes, followed by body mass and height measurements. Participants were then required to sit for 10 min before blood sampling. Whole blood samples were collected by venepuncture without venostasis from an antecubital vein using a 21G butterfly syringe into one lithium heparin (6 ml, 1.5 IU/ml heparin; Becton Dickinson, Oxford, UK) and one K_3_EDTA (6 ml, 1.6 mg/ml of K_3_EDTA; Becton Dickinson, Oxford, UK) vacutainer tube. Blood samples were immediately centrifuged and plasma aliquoted into eppendorfs and stored frozen initially at −20 °C during the ultramarathon events in a sterile field laboratory setup, prior to transferring to −80 °C storage after completion of the experimental procedure. Immediately after event completion (and each stage of the MSUM), blood samples were collected, followed by a mid-flow urine sample at the earliest convenience.

At the end of each competition day on stages 1 to 4 in the MSUM, and within the hour after event completion in the 24 h, trained researchers conducted a standardised interview on participants and support crews to ascertain all foods and fluids ingested, symptoms, and injuries. To improve dietary recording accuracy, before competition, participants and their support crews were educated and instructed to record in detail all foods and fluids ingested during the competition period (i.e. pre, during, immediately post (within 1 h of cessation), and rest periods, as per MSUM and 24 h relevance) in real time. Additionally, participants and support crews were instructed to keep food and beverage packaging of all foods and fluids consumed, which were collected by researchers.

Gastrointestinal symptoms and DI were explored through a symptomology tool and a medical log with physician verification. A Likert-type rating scale was used to quantify GIS, whereby symptoms were classified as indicated by ≥50 mm on a standard 100 mm visual analogue scale, with 0 mm indicative of no symptoms to 100 mm indicative of extreme symptoms resulting in event withdrawal. Severity of GIS was classified as low, moderate, and high based on the incidence of GIS reported (1 GIS, 2–3 GIS, and ≥4 GIS; respectively). Dermatological injuries were classified through visual identification. Severity of DI was also classified as low, moderate, and high based on the reported incidence of DI, the level of self-management and (or) medical intervention by the medical crew the injury received, associated pain severity (0 = no pain, 1 = minimal pain, 2 = mild pain, 3 = moderate pain, 4 = severe pain, and 5 = elimination from event), and evidence of established and progressive infection and inflammation through physician verification. Additionally, to determine carbohydrate adequacy, urine reagent strips (10SG urinalysis strips, Siemans Healthcare Diagnostic, NY, USA) were used to identify urinary ketones (i.e. acetoacetic acid) in pre- and post-running urine samples [12].

### Dietary Analysis

Food and fluid ingestion records from both ultramarathon events were used to calculate energy, nutrient, and water intake variables through Dietplan6 dietary analysis software (v.6.60, Forestfield Software, Horsham, West Sussex, UK). To improve the validity of the dietary analysis, all the nutritional information gathered from food and beverage packages were entered into the dietary analysis software program. In addition, to improve the reliability of the dietary analysis, all the completed dietary interview logs were blindly analysed by a second-trained researcher.

### Assessment of Hydration Status

Pre- and post-running plasma osmolality (P_Osmol_) was determined from 50 μl lithium heparin plasma samples in duplicate by freezepoint osmometry (Osmomat 030, Gonotec, Germany), as recommended previously [39]. Whole blood K_3_EDTA samples were used to determine haemoglobin concentration and haematocrit through a cell counter (Coulter ACT Diff, Beckham Coulter, USA), which were subsequently used to estimate changes in plasma volume (P_V_) relative to pre-competition values [40].

### Data Analysis

Data in the text, tables, and figures are presented as mean value ± standard deviation (SD), unless otherwise specified. Considering the potential influence of individual body mass differences on dietary intake variables, data analysis was performed on total values and adjusted for body mass [5, 6, 41]. Diagnostic checks (Shapiro–Wilks test of normality and Levene’s homogeneity of variance test) were performed before applying parametric statistics (SPSS v.22, IL, US). One-way ANOVA (or Kruskal–Wallis one-way) was applied to determine stage differences in GIS and DI incidence and impact of severity of symptoms and injuries on dependant variables. Significant main effects were analysed using post hoc Tukey’s HSD test. Independent-sample *t* test (or Mann–Whitney *U* test) was applied to determine dependant variable differences between groups (GIS and DI), including sub-group comparisons (running speed (slow ultramarathon runners: average speed <8 km/h and fast ultramarathon runners: average speed ≥8 km/h) and sexes). Significance was accepted at *p* < 0.05.

## Results

### Gastrointestinal Symptoms

**Fig. 1:**
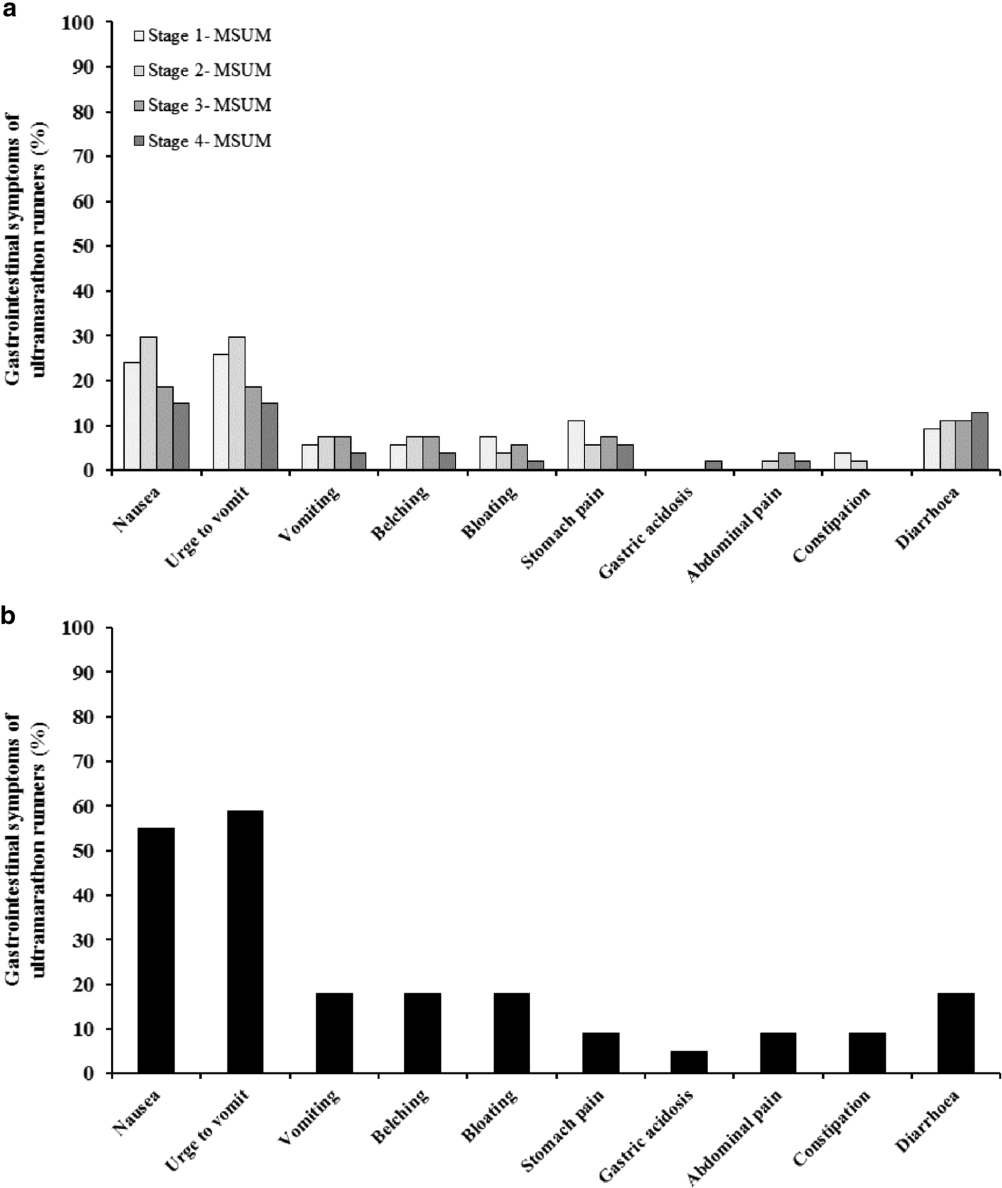
Gastrointestinal symptoms of ultramarathon runners during a multi-stage ultramarathon (**a**) and a 24-h continuous ultramarathon (**b**) (MSUM *n* = 54 and 24 h *n* = 22)

Gastrointestinal symptoms were a common feature during the MSUM, with 85 % of the study cohort presenting at least one GIS along the event (Fig. 1a). No statistically significant differences in GIS incidence or severity were observed between stages. No significant difference in GIS were observed between sexes. However, male ultramarathon runners reported greater occurrence (*p* = 0.04) of diarrhoea along the MSUM. Despite the presence of GIS being associated with a slower time to completion (28 h 43 min ± 4 h 32 min; 7.9 ± 1.3 km/h) compare with no-GIS (25 h 52 min ± 4 h 12 min; 8.9 ± 1.3 km/h), no significant difference in GIS was observed for running speed.

Gastrointestinal symptoms were a common feature during the 24 h, with 73 % of the study cohort presenting at least one GIS during the event (Fig. 1b). No significant differences in GIS were observed between sexes. Gastrointestinal symptoms were greater in fast (2.5-fold) compared with slow ultramarathon runners (*p* < 0.01). Fast ultramarathon runners reported greater occurrence of urge to vomit (*p* = 0.04), vomiting (*p* = 0.04), belching (*p* = 0.04), and diarrhoea (*p* = 0.04) compared with slow ultramarathon runners. On the contrary to the MSUM, the presence of GIS was not associated with a lower distance covered over the 24 h (166 ± 28 km; 6.9 ± 1.2 km/h) compare with no-GIS (139 ± 15 km; 5.8 ± 0.6 km/h).

### Dermatological Injuries

**Fig. 2:**
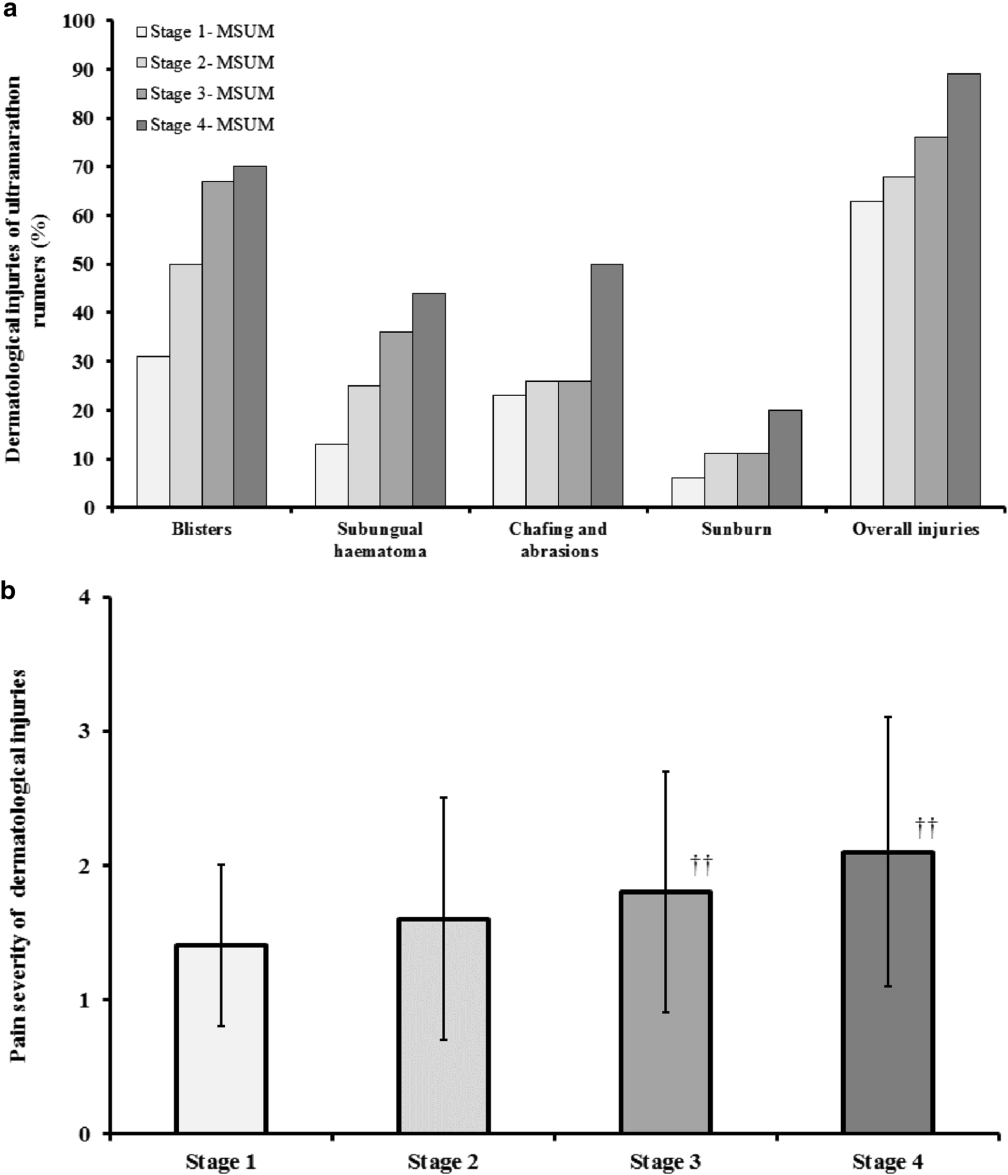
Dermatological injuries (**a**) and pain severity of dermatological injuries (**b**) of ultramarathon runners during a multi-stage ultramarathon. Pain severity scale: 0 = no pain, 1 = minimal pain, 2 = mild pain, 3 = moderate pain, 4 = severe pain, and 5 = elimination from event. Mean ± SD (*n* = 54): ^††^ *p* < 0.01 vs. Stage 1

Throughout the MSUM, 89 % of participants presented at least one DI, irrespective of severity. However, 76 % of participants required self-management and (or) sought (and received) medical intervention for DI. The incidence (*p* < 0.01) and severity (*p* < 0.01) of DI significantly increased as the MSUM progressed. No significant difference in DI incidence was observed between sexes and running speed. However, the presence of DI were associated within a slower time to completion (29 h 03 min ± 4 h 39 min; 7.8 ± 1.3 km/h) compare with no-DI (26 h 38 min ± 4h39min; 8.7 ± 1.3 km/h). Dermatological injuries that required self-management and (or) medical attention during the 24 h was low (14 %); thus, comparative analyses on intake and status variables were not possible (Fig. 2).

### Hydration Status

No statistically significant difference in pre- and post-stage P_Osmol_ (coefficient of variation: 3.5 %) was observed between participants presenting GIS and no-GIS in the MSUM (overall mean 286 ± 8 mOsmol/kg) and the 24 h (overall mean 286 ± 11 mOsmol/kg) and between participants presenting DI and no-DI in the MSUM (overall mean: 287 ± 7 mOsmol/kg). Similarly, no statistically significant difference in P_V_ change was observed between participants presenting GIS and no-GIS in the MSUM (peak value 21 ± 12 %) and the 24 h (peak value 9 ± 11 %) and between participants presenting DI and no-DI in the MSUM.

### Energy Balance

**Fig. 3:**
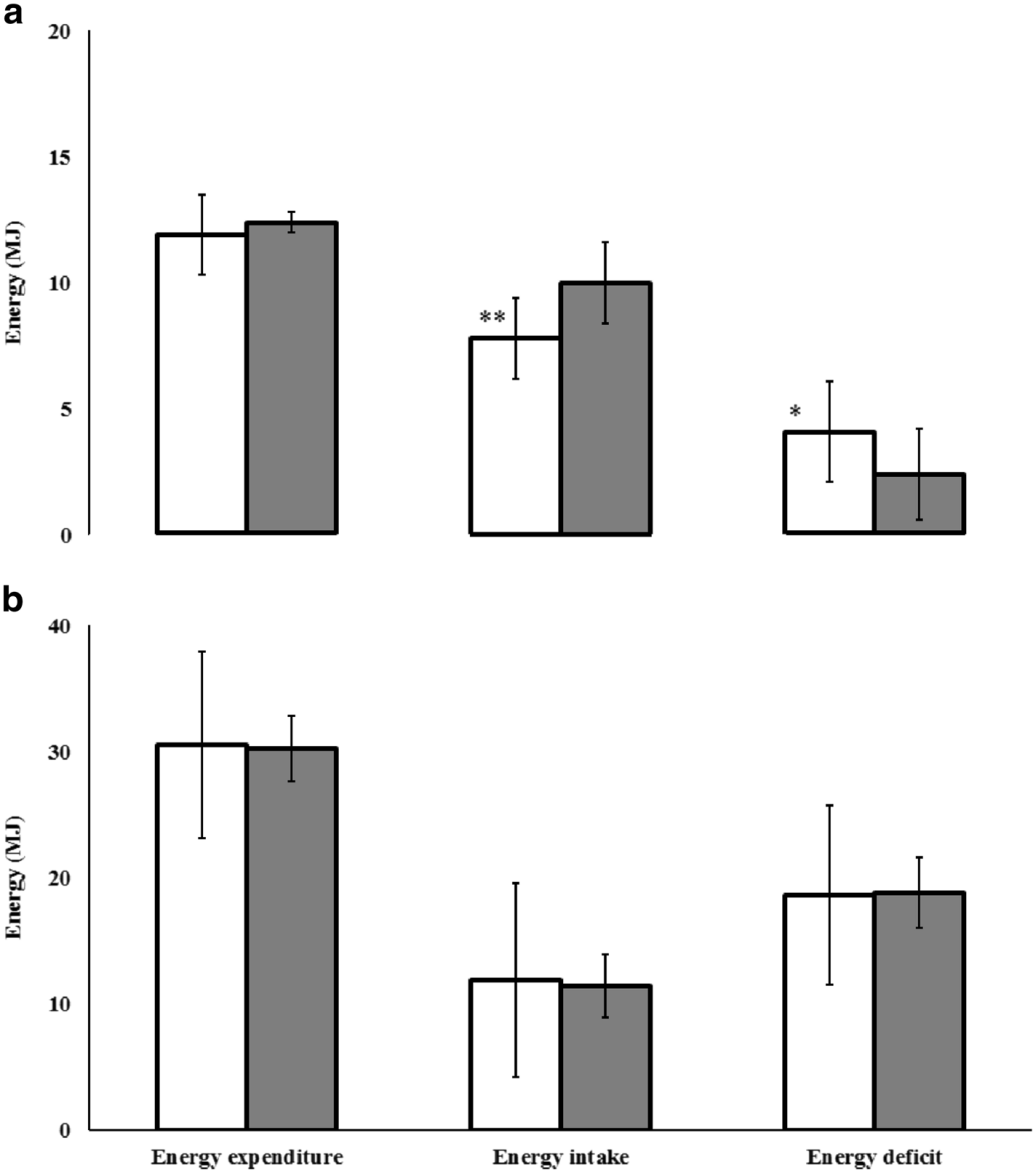
Energy expenditure, intake, and deficit of ultramarathon runners presenting (*white*) and not presenting (*grey*) gastrointestinal symptoms during a multi-stage ultramarathon (**a**) and a 24-h continuous ultramarathon (**b**). Mean ± SD (MSUM *n* = 54 and 24 h *n* = 22): **p < 0.01 and *p < 0.05 vs. no-GIS

Gastrointestinal symptoms during the MSUM were associated with a lower daily energy intake (total *p* < 0.01 and adjusted *p* = 0.03) and greater energy deficit (78 %; *p* = 0.04) compared with no-GIS (Fig. 3a). The severity of GIS did not further disturb energy balance along the MSUM. No significant differences in energy intake (total and adjusted), energy expenditure, and energy deficit were observed between GIS and no-GIS in the 24 h (Fig. 3b). Gastrointestinal symptoms along the MSUM were also associated with a reduction in energy intake during running compared with no-GIS (Table 2). Furthermore, greater GIS severity along the MSUM was associated with a lower energy (total (−1.5 MJ) *p* = 0.01 and adjusted *p* = 0.01) intake during running. Additionally, GIS along the MSUM were associated with a lower energy intake during the recovery period (Table 2); however, severity of GIS did not significantly exacerbate the reduction.

**Table 2:** During running and post-stage recovery energy, macronutrient, and water (through foods and fluids) intake of ultramarathon runners presenting (GIS) and not presenting (no-GIS) gastrointestinal symptoms during a multi-stage ultramarathon (MSUM)

	During			Post-stage recovery		
	GIS	no-GIS	*p* value	GIS	no-GIS	*p* value
Energy (MJ)						
Total	3.1 ± 2.0	4.0 ± 1.5	<0.05	2.4 ± 1.1	3.4 ± 1.4	<0.01
Adjusted^a^	0.046 ± 0.003	0.052 ± 0.002	0.36	0.036 ± 0.002	0.043 ± 0.002	0.07
Protein (g)						
Total	13 ± 28	13 ± 9	0.91	14 ± 14	23 ± 13	<0.01
Adjusted^a^	0.2 ± 0.4	0.2 ± 0.1	0.89	0.2 ± 0.2	0.3 ± 0.2	<0.05
Carbohydrate (g)						
Total	137 ± 75	175 ± 69	<0.05	108 ± 50	141 ± 87	0.07
Adjusted^a^	2.0 ± 0.9	2.3 ± 1.0	0.14	1.6 ± 0.8	1.6 ± 0.7	0.77
Poly (%)^b^	19 ± 16	26 ± 15	0.10	18 ± 21	10 ± 16	0.49
Oligo/di/mono (%)^b^	81 ± 16	74 ± 15	0.06	82 ± 21	90 ± 16	0.07
Fibre	3 ± 5	6 ± 4	<0.01	3 ± 3	3 ± 3	0.96
Fat (g)						
Total	15 ± 16	21 ± 15	0.06	10 ± 11	18 ± 13	<0.01
Adjusted^a^	0.2 ± 0.2	0.3 ± 0.2	0.34	0.2 ± 0.2	0.2 ± 0.2	<0.05
Water						
Total (L)	4.4 ± 1.5	3.9 ± 1.7	0.13	1.1 ± 0.6	1.5 ± 0.9	0.17
Adjusted (ml)^a^	65 ± 23	51 ± 22	<0.01	18 ± 10	20 ± 14	0.52

Mean ± SD (*n* = 54). The overall mean inter-observer coefficient of variation for energy, macronutrient, and water variables analysed were 1.3, 2.3, and 0.7 %, respectively

^a^Adjusted for body mass

^b^Saccharides

The presence and severity of DI along the MSUM did not result in significant differences of daily energy balance variables compared with no-DI. Incidence of DI did not significantly impact on energy intake during running (Table 3); however, greater severity of DI along the MSUM was associated with a lower energy (total (−1.2 MJ) *p* = 0.02 and adjusted *p* = 0.01) during running. Incidence and severity of DI did not significantly impact on energy intake in the post-stage recovery period (Table 3).

**Table 3:** During running and post-stage recovery energy, macronutrient, and water (through foods and fluids) intake of ultramarathon runners presenting (DI) and not presenting (no-DI) dermatological injuries during a multi-stage ultramarathon (MSUM)

	During			Post-stage recovery		
	DI	no-DI	*p* value	DI	no-DI	*p* value
Energy (MJ)						
Total	3.2 ± 2.2	3.2 ± 1.3	0.99	2.4 ± 1.2	2.6 ± 1.1	0.31
Adjusted^a^	.046 ± .031	.048 ± .021	0.63	.036 ± .018	0.040 ± 0.017	0.15
Protein (g)						
Total	13 ± 29	13 ± 12	0.93	14 ± 14	18 ± 15	0.07
Adjusted^a^	0.2 ± 0.4	0.2 ± 0.2	0.79	0.2 ± 0.2	0.3 ± 0.2	<0.05
Carbohydrate (g)						
Total	138 ± 77	151 ± 66	0.29	107 ± 51	115 ± 48	0.34
Adjusted^a^	1.9 ± 0.9	2.3 ± 1.0	<0.05	1.6 ± 0.8	1.7 ± 0.7	0.27
Poly (%)^b^	20 ± 17	19 ± 14	0.59	16 ± 19	20 ± 23	0.78
Oligo/di/mono (%)^b^	80 ± 17	81 ± 14	0.10	84 ± 19	80 ± 23	0.21
Fibre	4 ± 5	3 ± 2	0.11	3 ± 3	3 ± 5	0.75
Fat (g)						
Total	16 ± 18	14 ± 10	0.38	11 ± 11	13 ± 12	0.19
Adjusted^a^	0.2 ± 0.3	0.2 ± 0.1	0.76	0.2 ± 0.2	0.2 ± 0.2	0.25
Water						
Total (L)	4.5 ± 1.6	4.1 ± 1.1	0.05	1.1 ± 0.7	1.2 ± 0.6	0.75
Adjusted (ml)^a^	65 ± 24	61 ± 19	0.31	18 ± 11	18 ± 9	0.96

Mean ± SD (*n* = 54). The overall mean inter-observer coefficient of variation for energy, macronutrient, and water variables analysed were 1.3, 2.3, and 0.7 %, respectively

^a^Adjusted for body mass

^b^Saccharides

No acetoacetic acid was identified in pre-stage urine samples throughout the MSUM and pre-event urine samples in the 24 h. Acetoacetic acid (concentration range 1.5 to 8.0 mmol/l) in post-stage urine samples was evident in 46 % of participants at some point along the MSUM and 90 % of participants in the 24 h. No significant differences in pre- and post-stage acetoacetic acid concentration were observed between GIS and no-GIS and DI and no-DI along the MSUM. No significant differences in pre- and post-event acetoacetic acid concentration were observed between GIS and no-GIS in the 24 h.

### Macronutrient and Water Intake (Gastrointestinal Symptoms vs. No-Gastrointestinal Symptoms)

The presence of GIS during the MSUM was associated with a significant reduction in daily carbohydrate and fat intakes, compared with no-GIS (Table 4). Conversely, higher daily water intake was observed in participants reporting GIS (vs. no-GIS). The severity of GIS along the MSUM did not result in substantial differences of daily macronutrient intake. However, greater GIS severity did result in lower daily water intake (total (−851 ml/day) *p* = 0.04 and adjusted *p* < 0.01).

**Table 4:** Daily macronutrient and water (through foods and fluids) intake of ultramarathon runners presenting and not presenting (no-) gastrointestinal symptoms (GIS) and dermatological injuries (DI) during a multi-stage ultramarathon

	GIS	no-GIS	*p* value	DI	no-DI	*p* value
Protein (g)						
Total	104 ± 39	114 ± 28	0.20	106 ± 39	103 ± 38	0.68
Adjusted	1.5 ± 0.6	1.4 ± 0.3	0.46	1.5 ± 0.5	1.6 ± 0.6	0.61
Carbohydrate (g)						
Total	506 ± 137	635 ± 124	<0.01	516 ± 138	535 ± 151	0.39
Adjusted^a^	7.4 ± 2.0	7.9 ± 1.5	0.25	7.4 ± 1.8	7.8 ± 2.3	0.17
Poly (%)^b^	41 ± 11	35 ± 10	0.13	40 ± 11	39 ± 11	0.70
Oligo/di/mono (%)^b^	59 ± 11	65 ± 10	<0.01	60 ± 11	61 ± 11	0.37
Fibre	17 ± 9	29 ± 12	<0.01	18 ± 9	20 ± 12	0.35
Fat (g)						
Total	90 ± 36	126 ± 57	<0.01	95 ± 42	89 ± 36	0.33
Adjusted^a^	1.3 ± 0.6	1.6 ± 0.7	<0.05	1.4 ± 0.6	1.3 ± 0.5	0.62
Water						
Total (L)	7.8 ± 2.0	7.1 ± 2.2	0.09	7.9 ± 1.9	7.1 ± 2.1	<0.05
Adjusted (ml)^a^	117 ± 32	93 ± 31	<0.01	116 ± 31	109 ± 37	0.20

Mean ± SD (*n* = 54). The overall mean inter-observer coefficient of variation for energy, macronutrient, and water variables analysed were 1.3, 2.3, and 0.7 %, respectively

^a^Adjusted for body mass

^b^Saccharides

**Fig. 4:**
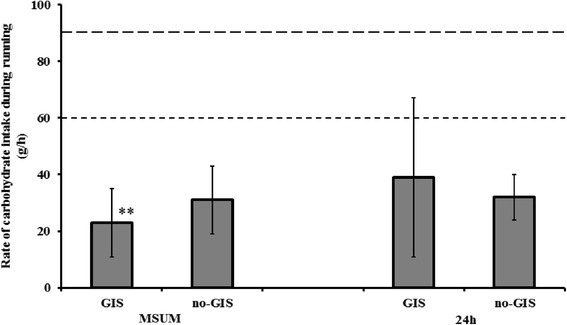
Rate of carbohydrate intake during running of ultramarathon runners presenting (*GIS*) and not presenting (no-GIS) gastrointestinal symptoms during a multi-stage ultramarathon (MSUM) and a 24-h continuous ultramarathon (24 h). Values compared to carbohydrate intake recommendation for glucose (*short broken line*) and glucose-fructose (*long broken line*) [43]. Mean ± SD (MSUM *n* = 54 and 24 h *n* = 22): **p < 0.01 vs. no-GIS

Incidence and severity of GIS during the 24 h did not alter macronutrient and water intake variables (total and adjusted) during running (Table 5); whereas GIS along the MSUM were associated with significant reductions in carbohydrate and fat intakes during running compared with no-GIS (Table 2). Rate of carbohydrate intake during running was also significantly lower (−8 g/h) in participants reporting GIS (*p* < 0.01 vs. no-GIS) (Fig. 4). Conversely, higher water intake during running was observed in participants reporting GIS (vs. no-GIS). Furthermore, greater GIS severity along the MSUM was associated with significantly lower protein (total (−19 g) *p* = 0.01 and adjusted *p* = 0.01), fat (total (−11 g) *p* = 0.03 and adjusted *p* = 0.01), and water (total (−902 ml) *p* = 0.01, adjusted *p* < 0.01, and rate per hour (−122 ml/h) *p* = 0.01) intakes during running.

**Table 5:** Macronutrient and water (through foods and fluids) intake of ultramarathon runners presenting and not presenting (no-) gastrointestinal symptoms (GIS) during a 24-h continuous ultramarathon

	GIS	no-GIS	*p* value
Protein (g)			
Total	79 ± 31	109 ± 49	0.12
Adjusted^a^	1.3 ± 0.4	1.3 ± 0.5	0.46
Carbohydrate (g)			
Total	937 ± 683	759 ± 184	0.54
Adjusted^a^	12.4 ± 8.7	8.7 ± 1.7	0.29
Poly (%)^b^	31 ± 10	33 ± 18	0.60
Oligo/di/mono (%)^b^	69 ± 10	67 ± 18	0.56
Fibre	23 ± 10	22 ± 15	0.82
Fat (g)			
Total	104 ± 53	143 ± 47	0.15
Adjusted^a^	1.1 ± 0.7	1.7 ± 0.6	0.51
Water			
Total (L)	9.1 ± 4.2	9.1 ± 3.8	0.98
Adjusted (ml)^a^	125 ± 54	105 ± 44	0.45

Mean ± SD (*n* = 22). The overall mean inter-observer coefficient of variation for energy, macronutrient, and water variables analysed were 0.8, 1.4, and 0.5 %, respectively

^a^Adjusted for body mass

^b^Saccharides

Gastrointestinal symptoms along the MSUM were associated with a reduced recovery nutrition profile, whereby significantly lower total protein, carbohydrate, and fat intakes were observed during the post-stage recovery period compared with no-GIS (Table 2). The severity of GIS along the MSUM did not however result in significant differences of macronutrient and water intake variables in the post-stage recovery period.

### Macronutrient and Water Intake (Dermatological Injuries vs. No-Dermatological Injuries)

Dermatological injuries along the MSUM did not result in substantial differences of daily macronutrient intakes compared with no-DI (Table 4). However, the presence of DI was associated with significantly higher daily water intake. Greater severity of DI along the MSUM was associated with significantly lower daily fat intake (total *p* = 0.01).

Dermatological injuries along the MSUM were associated with significant reductions in total protein and rate of carbohydrate (−4 g/h; *p* = 0.05; Fig. 4) intake during running compared with no-DI (Table 3). Conversely, higher water intake during running was observed in participants reporting DI (vs. no-DI). Greater severity of DI along the MSUM was associated with significantly lower carbohydrate (total (−40 g) *p* = 0.03 and rate per hour (−6 g/h) *p* = 0.02) intake and rate of water intake per hour (−105 ml/h; *p* = 0.02) during running.

The presence of DI along the MSUM did not significantly reduced recovery nutrition profile (Table 3). However, greater severity of DI along the MSUM was associated with significant reductions in the recovery nutrition profile, whereby lower protein (0.1 g/kg; adjusted *p* = 0.02) and water (total (−270 ml) *p* = 0.04 and adjusted *p* = 0.02) intakes were observed with increased severity.

## Discussion

The current study aimed to determine whether GIS or DI affects food and fluid intake, and nutrition and hydration status, of ultramarathon runners during multi-stage and 24-h ultra-marathons. Gastrointestinal symptoms were a common feature in both the MSUM and 24 h; however, reported incidence of DI requiring self-management and (or) medical intervention were high during the MSUM, but relatively low during the 24 h. The presence of GIS appeared to be associated with greater disturbances to daily energy balance, reduced daily macronutrient intake, and reduced nutritional intake during running and recovery throughout the MSUM. Similarly, the presence of DI appeared to be associated with reduced nutritional intake during running and recovery throughout the MSUM. Increasing severity of GIS and DI along the MSUM was associated with further reductions in energy, macronutrient, and fluid intakes. Gastrointestinal symptom incidence and severity did not alter energy, macronutrient, and fluid intakes during the 24 h. From a performance perspective and considering previous research has identified performance issues with GIS and DI [18], these clinical manifestation resulted in a longer time to complete the MSUM, but not distance covered in the 24 h, despite not reaching significance possibly due to large individual variations within group comparisons.

In both events, upper-GIS and nausea were the most commonly reported symptoms, with lower-GIS (i.e. predominantly diarrhoea) incidence being reported to a lesser extent. These results are in accordance with previous field observations, whereby 89 % of ultramarathon runners presenting gastrointestinal distress during a 161-km ultramarathon also reported nausea, whilst abdominal cramping (44 %) and diarrhoea (44 %) were reported to a lesser extent [17, 18]. Such observations are however contrary to previously reported indications that lower-GIS are the predominant GIS during running [14]. Moreover, incidence of GIS were greater than those previously reports during long distance triathlon events, marathon, and ultramarathon running [14, 17]. This finding is not surprising, considering the greater exertional and thermoregulatory strain compared with previous studies. It is unfortunate that on this occasion, the timing or distance at which symptoms occurred was not determined. If ultramarathon runners presented symptom later, after running onset, it would be expected to have lesser impact on nutritional intake than if symptoms were presented earlier. Considering the field setting of the study and the competitive nature of the events, the current study design per se did not allow symptom data to be collected during the actual competitive segment of each event. However, recent preliminary data from controlled laboratory trials has indicated GIS significantly increasing by 30 min (equivalent to 5.0-km distance point) and remained elevated thereafter, during a 3 h running protocol in thermoneutral condition (23.3 °C, 51 % RH) whereby participants consumed 30 g carbohydrate (2:1 glucose to fructose ratio) every 20 min [42, 43].

Taking into account that (a) no significant difference in GIS incidence and severity was seen between stages in the MSUM, (b) participants reported symptoms ‘coming and going’ along the consecutive days of competition, and (c) the variety of symptoms reported in both events; the precise causal mechanism/s for the high GIS incidence is not clear, but appears to be multi-factorial in origin. Exertion induced splanchnic hypoperfusion, ischemia, mechanical trauma, neuroendocrine stimulation, altered gastrointestinal motility, malabsorption, and altered epithelial permeability have all been directly or indirectly implicated [15, 20–26, 44]. It is however likely that the exertional heat stress exposure along the MSUM substantially contributed to the high GIS rates, since greater perturbations to gastrointestinal integrity (i.e. splanchnic hypoperfusion, splanchnic ischemia, and increase epithelial permeability) have been observed in heat stress experimental models (>30 °C ambient temperature) compared with thermoneutral conditions [21, 24, 45]. Indeed, lower perceptive tolerance rating to heat has been associated with greater reports of GIS in ultramarathon runners [5]. Moreover, daily and during running water intakes (through foods and fluids) were observed to be greater in ultramarathon runners reporting GIS. Since over-drinking behaviours and asymptomatic hyponatraemia were evident in the current MSUM event [41], it is possible that fluid intake strategies of ultramarathon runners may have also contribute to GIS [27–29], especially upper-GIS (e.g. gastric fluid load above individual tolerance), nausea, and vomiting (e.g. hyponatraemia). Gastrointestinal symptoms along the 24 h were likely to have been induced by the extreme duration of exertional stress, with or without feeding tolerance (i.e. forced feeding of scheduled dietary strategy) [15, 46, 47]. Similar to the 24 h findings, total carbohydrate intake rates have been reported to be positively correlated with nausea and GIS, but resulted in faster finishing times in long distance triathlon events [14]. Systemic endotoxaemic and cytokinaemic responses were observed in the current MSUM and 24 h events [25, 26]. Such responses have also been linked to GIS; therefore, exertional or exertional heat stress induced intestinal bacteria translocation and subsequent systemic inflammatory responses cannot be discounted as GIS promoters during ultramarathon events. On this occasion, during both the MSUM and 24 h, the type of carbohydrates consumed (i.e. quantity of highly resistant and fermentable carbohydrates associated with GIS [48]) did not differ between GIS and no-GIS. This suggests that gastrointestinal mechanisms and intake volumes are potentially responsible for GIS and not necessarily food and fluid choices. It is worth noting that excessive carbohydrate intakes during exercise above individually tolerated levels may directly contribute to the high occurrence of upper-GIS through neural and humoral-driven pathways stimulating the ileal break [30]. It is thus evident that GIS during ultramarathon running is multi-factorial. Conducting a series of controlled laboratory experiments to assess exertional stress (with and without heat stress) on gastrointestinal integrity and functional markers, and links to symptoms, would shed light on the key aetiological pathways of exertional induced upper- and lower-GIS.

Considering the already compromised nutrition status of ultramarathon runners during MSUM and 24 h events [5, 6], GIS further compromised the ability to consume energy, macronutrients, and fluid along the MSUM, but not during the 24 h. Indeed, compared with no-GIS, incidence of GIS were associated with (1) lower daily energy, carbohydrate, and fat intakes; (2) lower energy and carbohydrate intakes during running; and (3) poorer recovery nutrition throughout the MSUM. The severity of GIS appeared not to impact on total daily energy and macronutrient intakes, but high severity did result in a lower total daily water intake. The severity of GIS also appeared not to impact on recovery nutrition, but high severity did result in lower energy, protein, fat, and water intakes during running. Thus, the debatable question is whether symptoms per se limited the ability to consume foods and fluids or whether additional nutrient availability in the gastrointestinal tract prevented or suppressed symptoms. It has previously been shown that carbohydrate ingestion before and during exertion attenuated the reductions in post-exercise superior mesenteric artery and portal vein blood flow by Doppler method [49, 50]. This suggests that nutrient presence in the gastrointestinal tract may be protective against exertion induced splanchnic hypoperfusion and subsequent associated damage and symptoms [51].

The current study was the first to comprehensively assess DI during various ultramarathon events and its impact on nutritional intake and status. Dermatological injuries that warranted self-management and (or) medical intervention were a common feature during the MSUM; but relatively low incidence was observed in the 24 h. The discrepancy between the MSUM and 24 h is likely due to MSUM participants having sufficient time to manage DI, whilst stopping during the 24 h event would cost the participant valuable time. It is speculated that incidence of DI were likely to be substantially higher in the 24 h, but participants did not choose to investigate damage during the event. Nevertheless, incidence and severity of DI increased as the MSUM progressed. Foot injury (e.g. blisters) was the most common DI reported, with severity of foot injury associated with infection, local inflammation, and pain. Participants reported investing time and effort into self-management or seeking and receiving medical attention for injuries. This was especially apparent from Stage 3 onwards, in which medical crews reported observing concerning levels of infection and inflammation surrounding blisters in several participants.

Dermatological injuries were associated with a lower rate of carbohydrate intake during running, but the incidence of DI did not promote any additional decrements in other nutritional and hydration status variables per se compared with no-DI. However, as the severity of DI increased, lower energy, carbohydrate, and water intakes during running and lower protein and water intakes as part of recovery nutrition were observed. It thus appears, the greater the number of DI and subsequent progressive presentation of infection, inflammation, and pain, the more attention is focused towards the management and treatment of injuries, whilst nutrition was deprioritised.

From a performance and clinical perspective, carbohydrate energy substrate plays a crucial role in exercise performance [43, 52], fatigue attenuation [12], and immune competence [53] during periods of exertional stress. Sub-optimal daily and during exercise carbohydrate intake has been linked with hormonal (e.g. cortisol) and immune (e.g. cytokine profile and innate immune responses) dysfunction, potentially leading to states of chronic fatigue and depressed immunity [11, 12, 53, 54]. Additionally, carbohydrate, protein, and water play a fundamental role in recovery processes, with poor recovery nutrition being linked to reduced glycogen and tissue protein synthesis, hypohydration, and immunodepression [8, 9, 43, 53, 55, 56]. Indeed, positive correlations have been observed between energy and carbohydrate intakes with perceived recovery quality during a multi-stage ultramarathon event [5]. It is thus concerning that incidence and severity of both GIS and DI promoted further reductions in daily, during exercise, and recovery nutrition.

## Conclusions

High rates of GIS were reported in both events; however, incidence and severity of GIS only negatively altered nutritional intake during MSUM. Dermatological injury incidence and severity reduced nutrient intake during running and recovery in the MSUM. Considering the already compromised nutritional status of ultramarathon runners during multi-stage events, these findings demonstrate that GIS and DI further compromises intake. Effective GIS and DI prevention and management strategies should be investigated, especially in respect to consecutive days of exertional stress.

## Abbreviations

24 h: 24-h ultramarathon
DI: dermatological injuries
GIS: gastrointestinal symptoms
MSUM: multi-stage ultramarathon
P_Osmol_: plasma osmolality
P_V_: plasma volume
SD: standard deviation

## References

[1] Knoth C, Knechtle B, Rust CA, Rosemann R, Lepers R (2012). Participation and performance trends in multi-stage ultra-marathon- the ‘Marathon des Sables’ 2003–2012. Extreme Physiol Med..

[2] Murray A, Costa RJS (2012). Born to run. Studying the limits of human performance. BMC Med.

[3] Hoffman MD, Pasternak A, Rogers IR, Khodaee M, Hill JC, Townes DA (2014). Medical services at ultra-endurance food races in remote environments: medical issues and consensus guidelines. Sports Med.

[4] Krabak BJ, Waite B, Lipman G (2014). Evaluation and treatment of injury and illness in the ultramarathon athlete. Phys Med Rehabil Clin N Am.

[5] Costa RJS, Swancott A, Gill SK, Hankey J, Scheer V, Murray A (2013). Compromised energy and nutritional intake of ultra-endurance runners during a multi-stage ultra-marathon conducted in a hot ambient environment. Int J Sports Sci..

[6] Costa RJS, Gill SK, Hankey J, Wright A, Marczak S (2014). Perturbed energy balance and hydration status in ultra-endurance runners during a 24 h ultra-marathon. Br J Nutri..

[7] Scheer BV, Murray A (2011). Al Andalus Ultra Trail: an observation of medical interventions during a 219km, 5 day ultramarathon stage race. Clin J Sport Med..

[8] Costa RJS, Oliver SJ, Laing SJ, Walters R, Bilzon JLJ, Walsh NP (2009). Influence of timing of postexercise carbohydrate-protein ingestion on selected immune indices. Int J Sport Nutr Exerc Metab.

[9] Costa RJS, Walters R, Bilzon JLJ, Walsh NP (2011). Effects of immediate post-exercise carbohydrate ingestion with and without protein on neutrophil degranulation. Int J Sport Nutr Exerc Metab.

[10] Dempster S, Britton R, Murray A, Costa RJS (2013). Case study: nutrition and hydration status during 4254 km of running over 78 consecutive days. Int J Sport Nutr Exerc Metab.

[11] Robson-Ansley P, Barwood M, Canavan J, Hack S, Eglin C, Davey S (2009). The effect of repeated endurance exercise on IL-6 and sIL-6R and their relationship with sensations of fatigue at rest. Cytokine..

[12] Robson-Ansley PJ, Gleeson M, Ansley L (2009). Fatigue management in the preparation of Olympic athletes. J Sports Sci..

[13] Walsh NP, Gleeson M, Shephard RJ, Gleeson M, Woods JA, Bishop NC (2011). Position statement. Part one: immune function and exercise. Exerc Immunol Rev.

[14] Pfeiffer B, Stellingwerff T, Hodgson AB, Randell R, Pöttgen K, Res P (2012). Nutritional intake and gastrointestinal problems during competitive endurance events. Med Sci Sports Exerc..

[15] Rehrer NJ, McLaughlin J, Wasse LK, Maughan R (2014). Importance of gastrointestinal function to athletic performance and health. The encyclopaedia of sports medicine, an IOC medical commission publication: Sports Nutrition.

[16] Jeukendrup AE, Vet-Joop K, Sturk A, Stegen JH, Senden J, Saris WH (2000). Relationship between gastro-intestinal complaints and endotoxaemia, cytokine release and the acute-phase reaction during and after a long-distance triathlon in highly trained men. Clin Sci..

[17] Stuempfle KJ, Hoffman MD, Hew-Butler T (2013). Association of gastrointestinal distress in ultramarathoners with race diet. Int J Sport Nutr Exerc Metab..

[18] Hoffman MD, Fogard K (2011). Factors related to successful completion of a 161-km ultramarathon. Int J Sports Physiol Perform.

[19] Stuempfle KJ, Hoffman MD (2015). Gastrointestinal distress is common during a 161-km ultramarathon. J Sports Sci.

[20] ter Steege RW, Kolkman JJ (2012). The pathophysiology and management of gastrointestinal symptoms during physical exercise, and the role of splanchnic blood flow. Aliment Pharmacol Ther..

[21] van Wijck K, Lenaerts K, Grootjans J, Wijnands KA, Poeze M, van Loon LJ (2012). Physiology and pathophysiology of splanchnic hypoperfusion and intestinal injury during exercise: strategies for evaluation and preventions. Am J Physiol..

[22] Lambert GP (2009). Stress-induced gastrointestinal barrier dysfunction and its inflammatory effects. J Anim Sci..

[23] O’Connor FG, Casa DJ, Bergeron MF, Carter R, Deuster P, Heled Y (2010). American College of Sports Medicine round table on exertional heat stroke. Return to duty/return to play: conference proceedings. Curr Sports Med Rep.

[24] Yeh YJ, Law LY, Lim CL (2013). Gastrointestinal response and endotoxemia during intense exercise in hot and cool environments. Eur J Appl Physiol.

[25] Gill SK, Hankey J, Wright A, Marczak S, Hemming K, Allerton DM (2015). The impact of a 24-hour ultra-marathon on circulatory endotoxin and cytokine profile. Int J Sports Med.

[26] Gill SK, Teixeira A, Rama L, Rosado F, Hankey J, Scheer V (2015). Circulatory endotoxin concentration and cytokine profile in response to exertional-heat stress during a multi-stage ultra-marathon competition. Exerc Immunol Rev..

[27] Backer HD, Shopes E, Collins SL, Barkan H (1999). Exertional heat illness and hyponatremia in hikers. Am J Emerg Med.

[28] Hew-Butler T, Rosner MH, Fowkes-Godek S, Dugas JP, Hoffman MD, Lewis DP (2015). Statement of the third international exercise-associated hyponatremia consensus development conference, Carlsbad, California, 2015. Clin J Sport Med.

[29] Siegel AJ, d’Hemecourt P, Adner MM, Shirey T, Brown JL, Lewandrowski KB (2009). Exertional dysnatremia in collapsed marathon runners: a critical role for point-of-care testing to guide appropriate therapy. Am J Clin Pathol.

[30] Shin HS, Ingram JR (2013). McGill, Poppitt SD. Lipids, CHOs, proteins: can all macronutrients put a ‘brake’ on eating?. Physiol Behav.

[31] Lipman GS, Scheer BV (2015). Blisters: the enemy of the feet. Wilderness Environ Med.

[32] Lipman GS, Ellis MA, Lewis EJ, Waite BL, Lissoway J, Chan GK (2014). A prospective randomized blister prevention trial assessing paper tape in endurance distances (Pre-TAPED). Wilderness Environ Med.

[33] Scheer BV, Reljic D, Murray A, Costa RJS (2014). The enemy of the foot: blisters in ultra-endurance runners. J Am Podiatr Med Assoc.

[34] Krabak BJ, Waite B, Schiff MA (2011). Study of injury and illness rates in multiday ultramarathon runners. Med Sci Sport Exerc.

[35] Schwabe K, Schwellnus MP, Derman W, Swanevelder S, Jordaan E (2014). Less experience and running pace are potential risk factors for medical complications during a 56 km road running race: a prospective study in 26 354 race starters-SAFER study II. Br J Sports Med.

[36] American Dietetic Association, Dietitians of Canada, American College of Sports Medicine, Rodriguez NR, Di Marco NM, Langley S. American College of Sports Medicine position stand. Nutrition and athletic performance. Med Sci Sports Exerc. 2009;41(3):709–31. 10.1249/MSS.0b013e31890eb86 19225360

[37] American Dietetic Association, Dietitians of Canada, American College of Sports Medicine, Rodriguez NR, Di Marco NM, Langley S. American College of Sports Medicine position stand. Nutrition and athletic performance. Med Sci Sports Exerc. 2009;41(3):709–31. 10.1249/MSS.0b013e31890eb86 19225360

[38] Britton R, Dempster S, Moore JP, Costa RJS. The use of triaxial accelerometry to support dietary intervention during a multi-stage mountain ultra-marathon: a case study approach. J Sport Sci. 2011;29 Suppl 2:S132.

[39] Seifarth CC, Miertschischk J, Hahn EG, Hensen J (2004). Measurement of serum and plasma osmolality in healthy young humans—influence of time and storage conditions. Clin Chem Lab Med..

[40] Costa RJS, Crockford MJ, Moore JP, Walsh NP (2014). Heat acclimation responses of an ultra-endurance running group preparing for hot desert based competition. Eur J Sport Sci.

[41] Costa RJS, Teixiera A, Rama L, Swancott AJ, Hardy LD, Lee B (2013). Water and sodium intake habits and status of ultra-endurance runners during a multi-stage ultra-marathon conducted in a hot ambient environment: an observational study. Nutri J.

[42] Miall A, Khoo A, Rauch C, Gibson P, Costa RJS (2014). Repetitive gut challenge reduces gastrointestinal symptoms and malabsorption of carbohydrates during exertional stress. J Nutr Intermediary Metab..

[43] Burke LM, Hawley JA, Wong SHS, Jeukendrup AE (2011). Carbohydrates for training and competition. J Sports Sci..

[44] Peters HP, Akkermans LM, Bol E, Mosterd WL (1995). Gastrointestinal symptoms during exercise. The effect of fluid supplementation. Sports Med.

[45] Selkirk GA, McLellan TM, Wright HE, Rhind SG (2008). Mild endotoxemia, NF-kB translocation, and cytokine increase during exertional heat stress in trained and untrained individuals. Am J Physiol Regul Integr Comp Physiol..

[46] Van Nieuwenhoven MA, Brummer RM, Brouns F (2000). Gastrointestinal function during exercise: comparison of water, sports drink, and sports drink with caffeine. J Appl Physiol.

[47] Peters HP, Schep G, Koster DJ, Douwes AC, de Vries WR (1994). Hydrogen breath test as a simple noninvasive method for evaluation of carbohydrate malabsorption during exercise. Eur J Appl Physiol Occup Physiol.

[48] Halmos EP, Christophersen CT, Bird AR, Shepherd SJ, Gibson PR, Muir JG (2015). Diets that differ in their FODMAP content alter the colonic luminal microenvironment. Gut..

[49] Qamar MI, Read AE (1987). Effects of exercise on mesenteric blood flow in man. Gut.

[50] Rehrer NJ, Goes E, DuGardeyn C, Reynaert H, DeMeirleir K (2005). Effect of carbohydrate on portal vein blood flow during exercise. Int J Sports Med.

[51] Matheson PJ, Wilson MA, Garrison RN (2000). Regulation of intestinal blood flow. J Surg Res.

[52] Stellingwerff T, Cox GR (2014). Systematic review: carbohydrate supplementation on exercise performance or capacity of varying durations. Appl Physiol Nutr Metab..

[53] Walsh NP, Gleeson M, Pyne DB, Nieman DC, Dhabhar FS, Shephard RJ (2011). Position statement. Part two: maintaining immune health. Exerc Immunol Rev.

[54] Costa RJS, Jones GE, Lamb KL, Coleman R, Williams JH (2005). The effects of a high carbohydrate diet on cortisol and salivary immunoglobulin A (s-IgA) during a period of increase exercise workload amongst Olympic and Ironman triathletes. Int J Sports Med.

[55] Beelen M, Burke LM, Gibala MJ, van Loon LJC (2010). Nutritional strategies to promote postexercise recovery. Int J Sport Nutri Exerc Metab..

[56] American College of Sports Medicine, Sawka MN, Burke LM, Eichner ER, Maughan RJ, Montain SJ, et al. American College of Sports Medicine position stand. Exercise and fluid replacement. Med Sci Sports Exerc. 2007;39:377–90. 10.1249/mss.0b013e31802ca597 17277604

